# Surgical Treatment of Sacral Metastatic Tumors

**DOI:** 10.3389/fonc.2021.640933

**Published:** 2021-06-25

**Authors:** Mengxiong Sun, Dongqing Zuo, Hongsheng Wang, Jiakang Sheng, Xiaojun Ma, Chongren Wang, Pengfei Zan, Yingqi Hua, Wei Sun, Zhengdong Cai

**Affiliations:** Department of Orthopedics, Shanghai General Hospital, Shanghai, China

**Keywords:** sacral metastasis, bone tumor, surgery, radiofrequency ablation, sacroplasty

## Abstract

**Objective:**

This study intends to retrospectively analyze the data of patients with sacral metastases in our center, and analyze the treatment methods and therapeutic effects of sacral metastases.

**Methods:**

73 patients with sacral metastases treated in our hospital from June 2013 to June 2019 were retrospectively analyzed. There were 54 cases of neurological symptoms, 42 cases of sacroiliac joint instability, 24 cases of lower limb muscle weakness and 19 cases of abnormal urination and defecation. Four patients with tumors below S3 underwent complete tumor resection, 23 patients with tumors above S3 and without sacroiliac joint instability underwent tumor curettage and nerve root lysis, 34 patients with tumors above S3 and sacroiliac joint instability underwent tumor curettage, nerve root release and screw rod reconstruction. 12 patients with multiple metastases underwent percutaneous radiofrequency ablation and sacroplasty. VAS was used to evaluate the preoperative and postoperative pain scores, and the postoperative pain relief, neurological function, bowel function, wound healing and complications were evaluated.

**Results:**

There were no perioperative death, 8 cases of poor wound healing, 5 cases of nerve injury, postoperative sensory and motor loss of lower limbs. Cerebrospinal fluid (CSF) leak in 7 cases. The patients were followed up for 6-25 months (mean 12 months). The VAS scores of patients with pain symptoms were 7 points before operation and 1.44 points after operation, In 19 patients with abnormal urination and defecation function, 12 patients recovered to normal 3-6 months after operation, 5 cases had no significant change compared with preoperative, and 2 cases had aggravated symptoms; 17 cases of patients with lower limb muscle strength were significantly recovered after operation, and the average muscle strength was increased by 2 grades; 30 cases of patients with unstable sacroiliac joint got internal fixation had significantly pain relief. Pain symptoms of 9 patients were significantly relieved after percutaneous radiofrequency ablation.

**Conclusion:**

the operation of sacral metastases mainly adopts a relatively conservative surgical method, which can effectively improve the quality of life of patients with sacral metastases by retaining the nerve function and relieving the pain of patients, combining with radiofrequency ablation, sacroplasty and targeted drugs.

## Introduction

With the development of tumor treatment, many solid tumor patients can get effective treatment and obtain long-term survival. Some patients have bone metastasis in the late stage of treatment (1). Sacrum is also the site of bone metastases in tumor patients (2, 3). Because of the special anatomical position and abundant blood supply of sacrum (4), the treatment of sacral metastatic tumors is relatively difficult. At present, there is no unified standard and guideline for the treatment of sacral metastatic tumors in the world.

Most of the traditional treatments for sacral metastases are local radiotherapy or palliative treatment (2, 5). However, radiotherapy can alleviate the symptoms of patients with sacral metastases, but also has some limitations. For example, radiotherapy may bring wound complications, and due to the special location of the sacrum, radiotherapy may sometimes bring intestinal damage, resulting in radiation enteritis, and so on, and some tumors, due to multiple metastases, cannot solve all the problems through radiotherapy. At the same time, because of the low incidence rate, most cases reported sacral metastases are few (6). At present, the treatment and complications of sacral metastases are still not very clear.

In this study, the data of patients with sacral metastases in our center were analyzed retrospectively, including gender, age, primary tumor, symptoms, treatment methods, symptom remission, improvement of VAS score and complications.

## Materials and Methods

Data of 73 patients with sacral metastases from June 2013 to June 2019 were collected. The whole project was approved by the ethics committee of Shanghai General Hospital, and all patients signed informed consent. The general information of patients, primary tumor, local and systemic symptoms, treatment methods, pain relief, nerve function, defecation and other data were collected. VAS score system was used for pain relief before treatment, 1 month and 3 months after treatment, and ECoG score system was used for general condition of patients. According to the time nodes (3 months, 6 months, 9 months, 12 months, 24 months), the local control rate and overall survival time were collected. All patients were operated from the posterior approach. Subtotal resection is mainly used when the tumor invades the surrounding nerve seriously and needs to preserve the sacral nerve. It mainly uses curettage or piece-meal resection to preserve the sacral nerve to the maximum extent. En bloc resection is mainly used for complete resection of tumors, mainly for tumors below S3, and complete resection of tumors along the tumor edge.

### Sub-total Resection

When the tumor is located in S1-S2, in order to preserve the S1-S2 nerve root of the patient, we used sub-total resection. The specific implementation steps are to open the sacral lamina, decompress the nerve root, scrape off the tumor in the sacral vertebra, and further decompress the nerve root.

### En-Bloc Resection

When the tumor is located below S3, we used en-bloc resection. The specific implementation steps are: after exposing the tumor, we start en-bloc resection of the sacrum from the sacrum above the tumor to completely remove the tumor. During the operation, we should pay attention not to injure the intestine in front.

### Reconstruction

For patients with stable sacroiliac joint, we usually use bone cement sacroplasty for reconstruction after tumor resection, while for patients with unstable sacroiliac joint, we usually use spinal internal fixation screw rod for fixation.

### Statistical Analysis

All statistical analyses were performed using SPSS software package version 16.0 (SPSS Inc., Chicago, IL, USA). Paired t tests were used to assess the significance of the difference between the preoperative, 1-month postoperative and 3-month postoperative scores. The Kaplan–Meier method was used to estimate local control rates and overall survival rates. The log–rank test was used to compare the factors affecting local control. A p value < 0.05 was considered statistically significant.

## Results

A total of 73 patients were enrolled, including 33 males (45.2%) and 40 females (54.8%). The average age was 63 years old. 52 patients (71.2%) were over 60 years old and 21 patients (28.8%) were under 60 years old. The average follow-up time was 12 months (range, 6-25 months) (**Table 1**). In the early stage of the disease, 54 patients had lower limb pain, 42 patients had sacroiliac joint instability, 24 patients had lower limb muscle strength decline, 12 patients had urination dysfunction, and 7 patients had bowel dysfunction. The average duration of preoperative symptoms was 3 months (1-20 months) (**Table 2**). Among the 19 patients with abnormal urination or bowel function, 12 cases recovered to normal 3-6 months after operation, 5 cases had no obvious change compared with that before operation, and 2 cases had aggravation of symptoms; 17 cases of patients with lower limb muscle strength were significantly recovered after operation, and the average muscle strength was increased by 2 grades.

**Table 1:** Patients information about the 73 sacral metastasis.

Characteristics	N (%)
Gender	
Male	33 (45.2)
Female	40 (54.8)
Age	
≥60	52 (71.2)
<60	21 (28.8)
Sacral site	
S1-S3	69 (94.5)
S4-SS	4 (5.5)
Primary tumor	
Lung cancer	13 (17.8)
Breast cancer	9 (12.3)
thyroid cancer	7 (9.6)
prostate cancer	9 (12.3)
renal cancer	16 (21.9)
haematological malignancy	3 (4.1)
nasopharyngeal carcinoma	1 (1.14)
rectal cancer	15 (20.5)

**Table 2:** Symptoms and signs of the 73 patients.

Symptoms and signs	N (%)
Leg pain or buttock pain	54 (73.9)
sacroiliac joint instability	42 (57.5)
Lower limb muscle weakness	24 (32.8)
neurogenic bladder dysfunction	12 (16.4)
neurogenic bowel dysfunction	7 (9.6)

Among the primary tumor diseases, clear cell renal cell carcinoma was the most common, 16 cases in total, accounting for 21.9%. Among the remaining cancers, 13 cases were lung cancer (17.8%), 9 cases were breast cancer (12.3%), 7 cases were thyroid cancer (9.6%), 9 cases were prostate cancer (12.3%), 3 cases were hematological system tumor (4.1%), 1 case was nasopharyngeal carcinoma (1.4%), and 15 cases were rectal cancer (20.5%) (**Table 1**). Except for rectal cancer, the rest of the tumors were hematogenous metastasis, and most of the rectum was considered as local implantation or infiltration because of its anatomical location close to the sacrum. In all patients, 39 cases (53.4%) had single metastasis, while 34 cases (46.5%) had multiple systemic metastasis. 37 patients had not been treated with primary disease before operation, 10 patients received surgery and chemotherapy at the primary site, 5 patients received surgery at the primary site and radiotherapy at the sacrum, 14 patients received surgery and targeted drug therapy at the primary site, 4 patients received immunotherapy, and 3 patients received local perfusion chemotherapy at the metastatic site.

**Figure 1:**
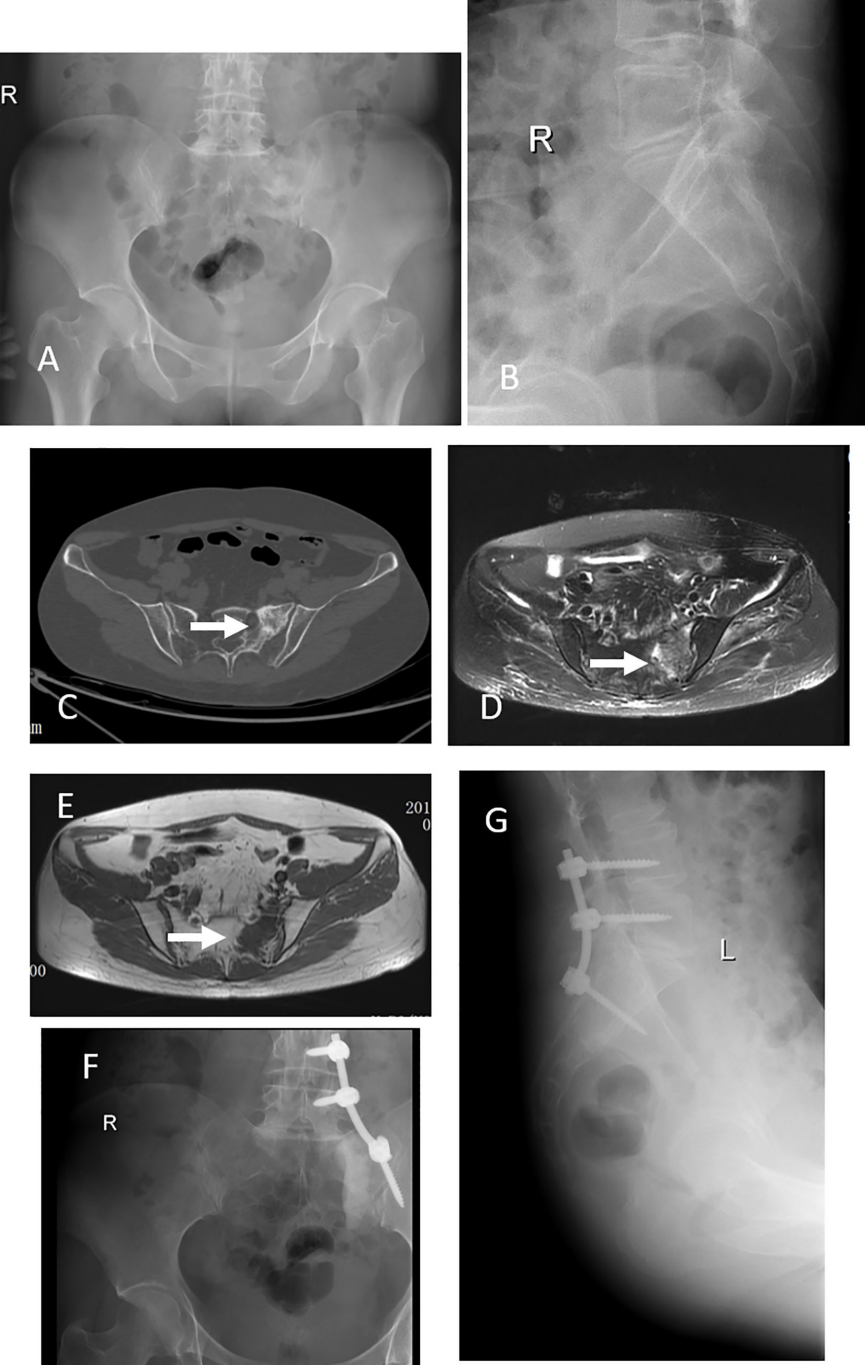
This 42 years old woman had severe back pain after breast cancer treatment for 7 years. X-ray and CT scan showed a tumor at left S1-2 **(A–C)**, an arrow indicate the lesion. MRI scan, both T1 and T2, indicated the tumor occurred in the left S1-2 **(D, E)**. This patient got a subtotal resection and pedicle screw fixation **(F, G)**.

In 69 patients, tumors were located at S1-S3 (94.5%), and in 4 patients, tumors were located at S4-S5 (5.5%) (**Table 1**). In the treatment, 4 patients with tumors below S3 were treated with en bloc resection. 23 patients with tumors above S3 and without sacroiliac joint instability underwent tumor curettage and neurolysis, 34 patients with tumors above S3 and sacroiliac joint instability underwent tumor curettage, nerve root release and Pedicle screw reconstruction (6), and 12 patients with multiple metastases underwent percutaneous radiofrequency ablation and sacroplasty (**Table 3**). 27 patients underwent preoperative embolization of tumor blood vessels (7). All operations were performed in prone position. Modified Galveston technology was used for internal fixation of sacroiliac joint.(**Figure 1**). Lung cancer and renal cancer patients continue to take targeted drugs after surgery (8, 9), and patients with nasopharyngeal carcinoma continue to receive radiotherapy (10). A patient with renal cell carcinoma recurred many times after resection of the sacral tumor for the first time. After the first operation, percutaneous radiofrequency ablation was used in the later several times due to the rich blood supply (11). The local swelling and pain symptoms of the patient were relieved after radiofrequency ablation. The pain of 30 patients with sacroiliac joint instability and lumbosacral internal fixation was significantly relieved, and the pain symptoms of 9 patients after percutaneous radiofrequency ablation and sacroplasty were significantly relieved.

**Table 3:** Treatment modality of the 73 patients.

Treatment modality	N (%)
Gross-total resection	4 (5.5)
Subtotal resection without fixation	23 (31.5)
Subtotal resection with fixation	34 (46.6)
percutaneous radiofrequency ablation + sacroplasty	12 (16.4)

None of the patients died during the perioperative period. There were 8 cases of poor wound healing, 5 cases healed after the first debridement, 2 cases healed after the second debridement, 1 case of wound sinus formation, 5 cases of nerve injury, postoperative sensory and motor loss of lower limbs, 1 case of partial recovery of nerve function during the follow-up period, and the remaining 4 cases did not recover significantly. Cerebrospinal fluid leakage occurred in 7 cases and healed after bed rest, head down and foot high position and wound pressure (12). Two cases of venous thrombosis of lower extremity were relieved after anticoagulation for 2 weeks (13). There were 3 cases of transient urinary retention, 2 cases of urinary tract infection, 1 case of rectal injury and 1 case of long-term internal fixation loosening. (**Table 4**)

**Table 4:** Complications of the 73 patients.

Complications	N
Poor wound healing	8
Wound infection	3
sinus tract	1
CSF leak	7
peripheral nerve injury	5
transient urinary retention	3
urinary tract infection	2
Internal fixation loosing	1
rectal injury	1
DVT	2

**Figure 2:**
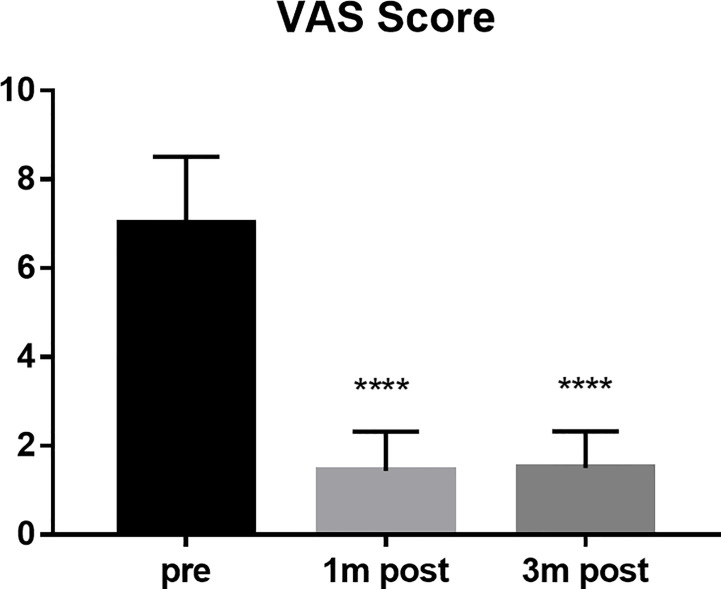
VAS score compared the scores of the patients pre, 1 month post and 3 months post operation. ****P < 0.0001.

**Figure 3:**
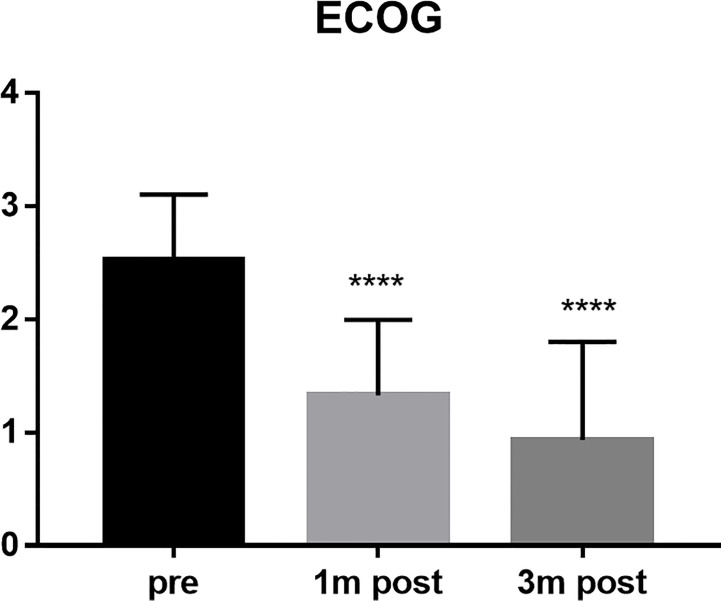
ECOG score compared the scores of the patients pre, 1 month post and 3 months post operation. ****P < 0.0001 and indicated the ECOG scores decreased after surgery.

The VAS scores of all patients were 7 points before operation, 1.44 points at 1 month after operation, and 1.51 points at 3 months after operation (P < 0.0001) (**Figure 2**). The average score of ECoG was 2.52 before operation, 1.33 at 1 month after operation and 0.93 at 3 months after operation (P < 0.0001) (**Figure 3**).

**Figure 4:**
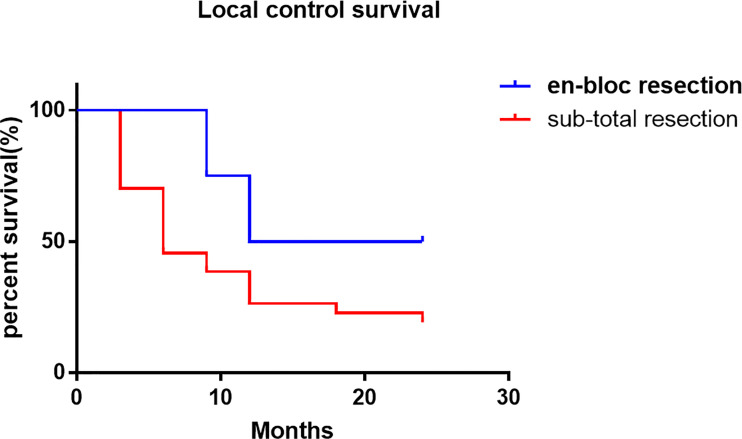
Local control survival rate compared the en-bloc resection and sub-total resection.

**Figure 5:**
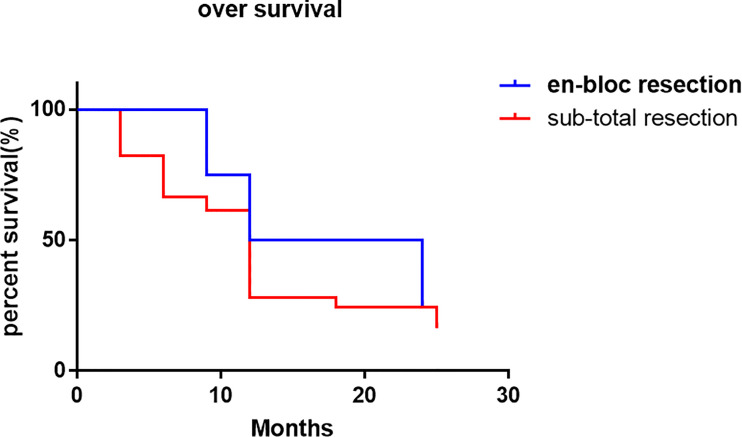
Overall survival rate compared the en-bloc resection and sub-total resection.

The local control time was 18 months (95% CI 0.72-12.36 months), and the local control rate was 75% and 50% at 9 months and 24 months, respectively. The average local control time of sub total resection group was 6 months (95% CI 0.81-1.37 months). The local control rates at 3 months, 6 months, 9 months, 12 months, 18 months and 24 months were 70.2%, 45.6%, 38.6%, 26.3%, 22.8% and 19.3%, respectively. There was no significant difference in the local control rate between the two surgical methods (P = 0.1515, log rank test). However, it can be seen from the survival curve that the two methods have a certain trend. En bloc resection seems to be more effective for local control (**Figure 4**). The overall survival rate of patients in en-bloc resection was 18 months (95% CI 0.46-4.82 months), and the overall survival rates of 9 months, 12 months and 24 months were 75%, 50% and 24.6%, respectively. The overall survival time of patients in sub total resection group was 12 months (95% CI 0.21-2.14 months). The overall survival rates of 3 months, 6 months, 9 months, 12 months, 18 months, 25 months were 82.5%, 66.7%, 61.4%, 28.1%, 24.6%, 16.4%, respectively. There was no significant difference in the overall survival rate between the two methods (P = 0.6653, log rank test) (**Figure 5**).

## Discussion

Sacral metastases are common tumors of the sacrum, but due to the existence of systemic diseases, the treatment of this metastatic site does not have a decisive impact on the overall survival time of patients. At present, palliative treatment is often used for the treatment of sacral metastases (14). The main purpose of palliative treatment is to relieve local symptoms, including pain and neurological dysfunction. The main treatment methods include some systemic medication and local radiotherapy. If a patient has a pathological sacral fracture and a risk of spinal cord compression, surgical intervention is often required in the case of local non-invasive treatment failure. In these cases, such as sacroiliac joint instability and pain, surgical intervention is particularly important. The contraindications of sacral metastases also include that the tumor situation of the whole body can not tolerate surgery or treatment. The multiple metastatic lesions of the whole body are often treated mainly by internal medicine, and the patients refuse surgery and other situations are not suitable for surgical treatment.

Although sacral metastases are common tumors in the sacrum, with the development of cancer treatment, many solid tumor patients can get a longer survival time (1). This also increases the incidence rate of bone metastases including sacral metastasis. However, the overall incidence of sacral metastases is still low, resulting in less reports on sacral metastases (15). There are unified norms and standards in the disease. This also increases the significance of our study.

The final prognosis of patients with sacral metastases is closely related to the pathological characteristics of patients with primary diseases. For example, in non-small cell lung cancer, gastrointestinal cancer and other tumors with poor response to systemic treatment (16, 17), although the sacral metastases have been removed, the overall prognosis is still poor. Therefore, the operation of sacral metastases should be as small as possible on the basis of nerve preservation functions. We had a case of lung cancer with sacral metastasis. When systemic chemotherapy drugs and local radiotherapy could not solve the pain caused by sacral lesions, lower limb dysfunction and defecation dysfunction, we gave him limited surgery and internal fixation, and the postoperative symptoms were well relieved. But in some primary diseases for breast cancer or prostate cancer patients with sacral metastases, this kind of patients have a good response of systemic treatment (18, 19). For this kind of patients, in order to solve the local pain and nerve symptoms caused by the metastasis, aggressive treatment modality should be involved, we had a prostate cancer metastasis patients, not only have sacral metastasis, but also There were bilateral proximal femur metastases. In order to solve the pain of the patient, we operated on all the sites, which causes pain in the patient, and continued to use endocrine therapy after the operation. The local symptoms were relieved of the patient and achieved a good prognosis. Another case is if the primary tumor is renal cell carcinoma. The response to systemic treatment of renal cell carcinoma is not very good, however, the prognosis is still good (20). Such patients should also be actively treated. We have a case of renal cancer patients with sacral metastasis, after the first sacral surgery, because the patient’s tumor blood supply is extremely rich, in order to reduce the intraoperative and postoperative complications, we conducted CT guided percutaneous radiofrequency ablation for the patient (21). The VAS score of the patients decreased significantly and the quality of life improved.

In this study, the vast majority of sacral metastatic tumors are located above S3. In order to preserve nerve function, en bloc is very difficult for tumors above S3. Therefore, extensive intralesional resection was performed in all these cases. In 4 cases, en bloc resection with preservation of all S1-S3 nerves was performed to maximize nerve function. Moreover, there was no local recurrence of sacrum in our 4 cases, which was the same as that reported by I. Feiz erfan et al. (15). Although en bloc resection can minimize local recurrence, in this group of S4-5 metastatic cases, after en-bloc resection, there are still S1-S3 recurrence or new metastases, which further proves that palliative surgery is still the main treatment for sacral metastases. However, although there is no statistical difference between en bloc resection and sub total resection in local control, there is still a certain trend in local control of en bloc resection, which can better control local recurrence. However, there is no significant difference between the two methods in the control of overall survival rate.

In terms of reconstruction after resection of sacrum tumor, sacroiliac joint instability is the absolute indication for screw rod reconstruction. In patients with pain due to sacroiliac joint instability before operation, after effective screw rod reconstruction, the pain symptoms of patients have been significantly relieved. In addition, in patients without sacroiliac joint instability, there is no significant difference between the two groups, Bone cement reconstruction can also significantly reduce postoperative pain. Therefore, effective sacroiliac joint fixation and bone cement molding can effectively reduce postoperative pain symptoms and improve the quality of life of patients.

Gaps in sacral operative area often lead to poor wound healing (22, 23). In this group of cases, there were 8 cases of poor wound healing, 5 cases of wound healing after the first debridement, 2 cases of wound healing after the second debridement, 1 case of wound sinus formation. This often needs the cooperation of plastic surgeons to solve the problem of poor wound healing. At the same time, minimally invasive surgery, such as percutaneous radiofrequency ablation, has a certain application space. Minimally invasive surgery can alleviate the local pain symptoms of patients (21), and will not cause wound complications. The incidence of wound complications will be increased if preoperative radiotherapy was performed. Whether a part of patients with sacral metastases can be considered for surgical treatment first, and then continue to use radiotherapy control in case of local recurrence or progression should also be considered (24).

However, this study also has some limitations. Firstly, as a retrospective study, there is no control group, so it is impossible to compare the priority and selectivity of non-surgical method and surgical method in the treatment of sacral metastases. Secondly, there is no separate list of primary pathological tumor species for single analysis, and it is impossible to analyze and judge the treatment methods, prognosis and complications of sacral metastases from the perspective of different primary disease. Therefore, it is necessary to conduct a randomized controlled study to analyze the control and complications of different treatment methods for sacral metastases. It is also necessary to expand the number of single primary pathological types of sacrum metastases for different studies

In conclusion, the treatment of sacral metastases is still based on the treatment of primary diseases, and palliative treatment is still the main course to relieve symptoms of sacral metastasis. This study retrospectively analyzed the treatment methods of sacral metastatic tumors, mainly focused on the surgical treatment of sacral metastases, analyzed the general situation and disease characteristics of patients with sacral metastases, analyzed the advantages and complications of surgery, radiofrequency ablation and other methods in sacral metastases, and concluded that en-bloc resection is somehow an effective method for the treatment of sacral metastases but have no significant influence on the over survival. Therefore, the treatment of sacral metastases still needs to be combined with the systemic treatment of the primary disease in order to obtain a better prognosis.

## Data Availability Statement

The original contributions presented in the study are included in the article/supplementary material. Further inquiries can be directed to the corresponding author.

## Ethics Statement

The project was approved by the ethics committee of Shanghai General Hospital, and all patients signed informed consent.

## Author Contributions

Conceptualization: MS and WS. Data curation: MS and DZ. Formal analysis: HW. Funding acquisition: MS, WS, YH, and ZC. Investigation: MS, WS, XM, and JS. Methodology: CW and PZ. Project administration: MS, WS, and ZC. Resources: MS and WS. Supervision: ZC. Validation: MS. Visualization: MS and WS. Writing—original draft: MS. Writing—review and editing: MS, WS, and ZC. All authors contributed to the article and approved the submitted version.

## Funding

Supported by National Natural Science Foundation of China (number: 8177101011).

## Conflict of Interest

The authors declare that the research was conducted in the absence of any commercial or financial relationships that could be construed as a potential conflict of interest.

## Abbreviation

VAS, Visual Analogue Scale; ECOG, Eastern Cooperative Oncology Group; CSF, Cerebrospinal fluid.
